# Heterogeneous effects of oil shocks on exchange rates: evidence from a quantile regression approach

**DOI:** 10.1186/s40064-016-2879-9

**Published:** 2016-07-27

**Authors:** Xianfang Su, Huiming Zhu, Wanhai You, Yinghua Ren

**Affiliations:** 1 College of Business Administration, Hunan University, Changsha, 410082 People’s Republic of China; 2 School of Economics and Management, Fuzhou University, Fuzhou, 350116 People’s Republic of China

**Keywords:** Oil shocks, Exchange rates, Heterogeneity effects, Quantile regression

## Abstract

The determinants of exchange rates have attracted considerable attention among researchers over the past several decades. Most studies, however, ignore the possibility that the impact of oil shocks on exchange rates could vary across the exchange rate returns distribution. We employ a quantile regression approach to address this issue. Our results indicate that the effect of oil shocks on exchange rates is heterogeneous across quantiles. A large US depreciation or appreciation tends to heighten the effects of oil shocks on exchange rate returns. Positive oil demand shocks lead to appreciation pressures in oil-exporting countries and this result is robust across lower and upper return distributions. These results offer rich and useful information for investors and decision-makers.

## Background

Oil is commonly regarded as a comparative advantage and key strategic resource and its prices dynamics can affect the real economy and financial markets. In international oil markets, because the US dollar is the major invoicing and settlement currency, the exchange rate quoted as foreign currency per US dollar is the primary channel through which an oil price shock is transmitted to the real economy and financial markets. Therefore, the impact of oil shocks on exchange rates is an important topic to investigate.

Since Golub (1983) and Krugman (1983) put forth arguments as to why movements in oil prices should affect exchange rates, numerous studies have examined the relationship between oil prices and exchange rates. Some studies present evidence of a weak nexus between oil price shocks and exchange rates. For instance, Huang and Guo (2007) suggest that real oil price shocks led to a minor appreciation in China’s real exchange rate. Basher et al. (2012) also examine the relationship between oil prices, exchange rates and stock prices, offering limited support for the relationship between oil prices and exchange rates. Reboredo (2012) finds that the dependence between oil prices and exchange rates is weak and there is no extreme market dependence between oil prices and exchange rates. On the other hand, more studies reveal that oil price shocks are the determinants of exchange rates. Amano and Van Norden (1998) conclude that the oil price causes persistent US dollar real exchange rate shocks. Using panel co-integration techniques, Camarero and Tamarit (2002) find that real oil price is one of the main determinants of the long-term real exchange rate for the Spanish peseta. Furthermore, there is considerable disagreement about whether the relationship between oil prices and exchange rates is positive or negative. A number of studies find a positive link between the oil price and the US dollar, meaning that an increase in the price of oil is associated with US dollar appreciation (e.g., Akram 2004; Rautava 2004; Benassy-Quere et al. 2007; Chen and Chen 2007; Coudert et al. 2008; Ghosh 2011; Cavalcanti and Jalles 2013). By contrast, some studies conclude that there is a depreciation of US dollar exchange rates following a rise in the price of oil (e.g., Narayan et al. 2008; Askari and Krichene 2010; Wu et al. 2012; Ji and Fan 2012; Aloui et al. 2013; Turhan et al. 2013).

From the previous discussion, it is safe to say that extant empirical evidence on the relationship between oil prices and exchange rates is mixed. From an econometric viewpoint, several reasons may explain the great discrepancies: the sample used for analysis, the model and the method employed to estimate the relationship. Moreover, we argue that these results may be biased given that the distributional heterogeneity of exchange rate returns is neglected. Theoretically, an oil price shock may be transmitted to a country’s exchange rate through two different channels: the terms of trade channel and wealth effect channel. The two distinct channels impact the exchange rate of oil-exporting and oil-importing countries differently.^1^ However, we primarily focus on the distributional heterogeneity of exchange rate responses to oil price shocks. It is necessary to discuss the theoretical considerations as to why the exchange rate responses would exhibit distributional heterogeneity. We believe that when the exchange rate of one country experiences large depreciation or appreciation, the terms of trade and current account balance will change, which subsequently leads to exchange rate responses exhibiting distributional heterogeneity. The “central bank intervention effect” and the “export selection effect”^2^ can partly explain the heterogeneous behaviour of exchange rate reactions. For example, according to the “central bank intervention effect” view, when a country’s currency experiences devaluation, in order to avoid the deteriorating effects of currency depreciation, the central bank will intervene and buy the specific currency (Beine et al. 2002; Wieland and Westerhoff 2005), which will consume the foreign exchange reserves and lead to a current account imbalance. According to the “export selection effect” view, when a currency experiences depreciation, higher performance firms tend to absorb exchange rate variations in their markup. For the highest decile in terms of size, exporters increase their export price by 2.5 % following a 10 % depreciation of the exchange rate (Berman et al. 2012). The behaviours of increasing export price will change the terms of trade and affect the responses of the exchange rate to oil price shocks.

In this paper, all exchange rates are quoted as foreign currency per US dollar. A positive exchange rate return hence implies an appreciation of the US dollar (right tail of the return distribution) and a negative return implies a depreciation of the US dollar (left tail of the return distribution).^3^ Consequently, lower return quantiles correspond to large US dollar depreciations, whereas upper return quantiles correspond to large US dollar appreciations. Given the existence of a “central bank intervention effect” and the “export selection effect”, different quantiles of the exchange rate return distribution associated with different terms of trade and current accounts should mitigate or amplify the impact of oil shocks on exchange rates. This is especially true, when the US dollar experiences large depreciation (e.g., at 0.01, 0.05, and 0.1 quantiles), i.e., indicating the local currency in large appreciation. The central bank would accumulate foreign exchange reserves to restrain appreciation pressures on a local currency. Therefore, the impact of oil price shocks on exchange rates would be mitigated by the accumulation of foreign exchange reserves which is unlike when the US dollar experiences large appreciation (e.g., at 0.90, 0.95, and 0.99 quantiles), i.e., indicating the local currency is experiencing large depreciation. Under the actions of the “central bank intervention effect” and “export selection effect”, the foreign exchange reserves will be reduced and the terms of trade will deteriorate, which would amplify the impact of oil price shocks on exchange rates. Therefore, the distributional heterogeneity of exchange rate returns should be taken into consideration when investigating the relationship between oil prices and exchange rates.

More recently, the empirical study on the impact of oil price shocks on exchange rates has evolved along multiple directions. On the one hand, many researchers consider the oil market characteristic and conclude that the response of exchange rates to oil shocks may be dependent on whether the country is oil importing or oil exporting (Lizardo and Mollick 2010; Fratzscher et al. 2014; Rasmussen and Roitman 2011). For example, Lizardo and Mollick (2010) show that increases in the real price of oil lead to a significant depreciation of the US dollar relative to the currency of oil-exporting countries such as Canada, Mexico and Russia, whereas an appreciation of the US dollar relative to the currency of oil-importing countries such as Japan. On the other hand, Kilian (2009) suggests that the impact of oil price shocks greatly depends upon whether the source of oil price fluctuations originates from an oil supply shock, a global aggregate demand shock or an oil-specific demand shock. Following Kilian (2009), some researchers study the impact of oil supply and demand shocks on exchange rates (Buetzer et al. 2012; Atems et al. 2015; Basher et al. 2016). In particular, Basher et al. (2016) use a two-stage approach to examine the impact of oil supply and demand shocks on real exchange rates. In the first step, they construct three oil structural shocks following Kilian (2009). In the second step, they use a two-regime (high- and low-volatility regime) Markov-switching model to investigate the nonlinear impact of oil shocks on real exchange rates. The Markov-switching model assumes that the exchange rate is governed by regimes that can be related to high volatility or low volatility states. However, the Markov-switching model is feasible for a small number of regimes, and cannot capture the entire distributional heterogeneity of exchange rate responses.

In this paper, we contribute to the literature by allowing for distributional heterogeneity for the effects of the oil supply and demand shocks on exchange rates for a set of representative oil-exporting and oil-importing countries: Australia, Canada, European Union, Japan, Mexico, Norway and the United Kingdom. The heterogeneous effect that we consider is associated with the various quantiles of exchange rate returns. We apply a quantile regression model to investigate the heterogeneous effects of the three oil shocks, constructed according to Kilian (2009), on exchange rates. By employing the quantile regression model we extend the earlier analysis by looking at the impact of oil shocks not only on the mean but also on the shape of the conditional distribution of exchange rate returns. In particular, we examine how the response of exchange rates for oil supply and demand shocks changes in periods of greater US dollar depreciation and appreciation. Are there different distributional heterogeneities in oil-exporting countries compared to oil-importing countries? Although it is necessary for investors and traders to capture the heterogeneous impact of oil shocks on exchange rates, to the best of our knowledge, no paper has yet thoroughly investigated whether US dollar depreciation or appreciation can dampen or amplify the impact of oil shocks on real exchange rates in a quantile regression framework.

The motivation to employ quantile regression on the shocks equation is twofold. On the one hand, quantile regression is able to describe the entire conditional distribution of exchange rate returns and thus help us obtain a more complete picture of the factors affecting oil price shocks. Specifically, the quantile regression estimator gives one solution to each quantile. Therefore, we may assess how oil shocks affect exchange rates according to their position on the conditional return distribution. Using this methodology, we are able to assess the determinants of shocks throughout the conditional distribution, with a particular focus on a large US dollar depreciation and appreciation periods that are arguably of greatest interest. From a risk perspective, it is more interesting to understand what happens at the extremes of a distribution. However, oil shock analyses using OLS techniques are not particularly suitable in a period of large US dollar depreciation and appreciation. In the quantile regression framework, the focus is no longer on the mean effect, but on the full distribution of exchange rate returns. Second, the quantile regression estimator is robust to outlying observations on the dependent variable and it can be more efficient than the OLS regression when the error term is non-normal. This is of particular advantage in oil shocks equation setting where the exchange rate return distributions are typically characterized by thick tails.

At present, a few papers have applied the quantile regression model to investigate the behaviour of exchange rates. For example, Nikolaou (2008) argues that the quantile regression approach allows us to directly capture the impact of different magnitudes of shocks that hit the real exchange rate, and can detect asymmetric, dynamic adjustment of the real exchange rate. Huang et al. (2011) find that the quantile regression approach generally produces more reliable exchange rate volatility forecasts than other key methods. Tsai (2012) uses the quantile regression model to investigate the relationship between stock price index and exchange rate in Asian markets, finding that the negative relation between stock and foreign exchange markets is more obvious when exchange rates are extremely high or low. Kuck et al. (2015) apply quantile regression techniques to investigate the temporal dependence patterns of exchange rates, and indicate that US dollar appreciations tend to feature positive dependence on past returns, while US dollar depreciations tend to feature negative dependence on past returns. These studies from different angles show that there is distributional heterogeneity on exchange rates across various quantiles. However, no paper has yet thoroughly investigated the heterogeneous effects of oil shocks on exchange rates in the quantile regression model framework.

The remainder of this paper is organized as follows. In “Methods” section, we discuss the empirical strategy, and in “Data and summary statistics” section, we describe the data and their descriptive statistics. “Empirical results” section provides the empirical results, and “Conclusions” section presents the study’s conclusions.

## Methods

### Identification of oil structural shocks

In order to identify these structural shocks in the oil market, a reduced-form VAR model specified as

1 $$y_{t} = \alpha + \sum\limits_{i = 1}^{p} {A_{i} } y_{t - i} + e_{t} ,$$

where $y_{t}$ is a 3 vector of endogenous variables, which includes the percentage change in global crude oil production, $\Delta prod$, an index of global real economic activity, $rea$, and the real price of oil, $rpo$. $\alpha$ is a 3 vector of intercept to be estimated. $A_{1} , \ldots ,A_{p}$ are matrices of coefficients to be estimated and $e_{t}$ is 3 vectors of innovation that may be contemporaneously correlated but are uncorrelated with their own lagged values and uncorrelated with all of the right-hand side variables. Note that a VAR mode containing a mix of stationary and non-stationary variables may suffer from a type of spurious regression problem. Thereby, we should pre-test our variables for stationarity when estimating the VAR model.

We assume that $e_{t}$ is related to the fundamental crude oil markets shocks $\varepsilon_{t}$ according to $e_{t} = A_{0}^{ - 1} \varepsilon_{t}$. Thus, Eq. (1) can be rewritten as a crude oil market shocks structural VAR model

2 $$A_{0} y_{t} = A_{0} \alpha + \sum\limits_{i - 1}^{p} {A_{0} A_{i} y_{t - i} + \varepsilon_{t} } .$$

Following Kilian (2009), $\varepsilon_{1t}$ denotes the oil supply shock, $\varepsilon_{2t}$ represents global aggregate demand shock, and $\varepsilon_{3t}$ is the oil-specific demand shock. Intuitively, we refer to them as the oil supply shock, aggregate demand shock and oil demand shock respectively. The identification of $A_{0}^{ - 1}$ is achieved by imposing the following exclusion restriction:

3 $$e_{t} = \left( \begin{array}{l} e_{1t}^{\Delta prod} \\ e_{2t}^{rea} \\ e_{3t}^{rpo} \end{array} \right) = \left[ \begin{array} {lll} a_{11} & 0 & 0 \\ a_{21} & a_{22} & 0 \\ a_{31} & a_{32} & a_{33} \end{array} \right] \left( \begin{array}{l} \varepsilon_{1t}^{oil\,supply\,shock} \\ \varepsilon_{2t}^{aggregate\,demand\,shock} \\ \varepsilon_{3t}^{oil\,demand\,shock} \end{array} \right).$$

The identifying restrictions underlying Eq. (3) first imply that the oil supply does not respond to oil demand or oil price shocks contemporaneously. This can be interpreted this way for the following reason: because of the high costs of changing production in the short run, oil producers are reluctant to change their output immediately following changes in demand. Secondly, oil demand changes in impact following an oil supply shock but not an oil price shock. This restriction is in line with the sluggish adjustment of global real economic activity due to movements in oil prices. Finally, the real oil price is assumed to respond to oil supply and demand shocks within the month. This assumption is plausible, as any exogenous changes in crude oil supply or the real economy are immediately reflected in oil prices.

### Quantile regression model of oil structural shocks

The impacts of oil shocks on each exchange rate can be estimated based on the linear regression model^4^:

4 $$R_{i,t} = \beta_{0,i} + \beta_{1,i} \varepsilon_{t}^{s} + \beta_{2,i} \varepsilon_{t}^{d} + \beta_{3,i} \varepsilon_{t}^{p} + u_{i,t} ,$$

where $R_{i,t}$ is the exchange rate return for country $i$ at time $t$. $\varepsilon_{t}^{s} ,{\kern 1pt} {\kern 1pt} {\kern 1pt} {\kern 1pt} \varepsilon_{t}^{d}$ and $\varepsilon_{t}^{p}$ are the identified oil supply, aggregate demand and oil-specific demand shocks respectively. This model does not include lags of the oil shocks as explanatory variables because exchange rate markets are very efficient and new information is quickly absorbed by the exchange rate market when the exogenous shock occurs.

In the linear regression model, the coefficients estimated by OLS are the conditional means of the model parameters. This conditional mean may capture how the real exchange rate varies with respect to oil market shocks in general, but it has limited informational value to reflect the impact of oil market shocks on real exchange rates during a period of US dollar depreciation or appreciation. In fact, it would be useful to understand how real exchange rates respond to oil market structural shocks at the tails of the exchange rate return distribution, which can be better revealed by quantile regression. Compared to the linear regression model, quantile regression provides a more precise and accurate result of the impact of conditional variables on the dependent variable.

The quantile regression approach provides specific insights on the impacts of oil market structural shocks on the real exchange rate returns under different market circumstances, including a large US dollar depreciation (lower quantile) and appreciation (upper quantile). Therefore, in order to account for the possible heterogeneous impact of oil market shocks on real exchange rates, a conditional quantile regression model relative to Eq. (4) is specified as

5 $$Q_{q} (R_{i,t} |x_{i,t} ) = x_{i,t}^{{\prime }} \cdot \beta_{i,q} ,$$

where $Q_{q} (R_{i,t} |x_{i,t} )$ denotes the *q*th conditional quantile of real exchange rate returns $R_{i,t}$ on the regressor vector $x_{i,t}$, $x_{i,t} = (1,{\kern 1pt} \,\varepsilon_{t}^{s} ,\,\varepsilon_{t}^{d} ,\,{\kern 1pt} \varepsilon_{t}^{p} )^{{\prime }}$, $\beta_{i,q} = (\beta_{i,0,q} ,\,\beta_{i,1,q} ,\,\beta_{i,2,q} ,\,\beta_{i,3,q} )^{{\prime }}$. The unknown parameter vector $\beta_{i,q}$ can be estimated for any quantile $q \in (0,{\kern 1pt} {\kern 1pt} {\kern 1pt} {\kern 1pt} 1)$ by minimizing the following expression with respect to $\beta_{i,q}$:

6 $$\hat{\beta }_{i,q} = \mathop {arg\,\hbox{min} }\limits_{{\beta_{i,q} \in R^{5} }} \sum\limits_{t = 1}^{T} {\left( {q - 1_{{\{ y_{i,t} < x_{i,t}^{{\prime }} \beta_{i,q} \} }} } \right)\,\left| {R_{i,t} - x_{i,t}^{{\prime }} \beta_{i,q} } \right|} ,$$

where $1_{{\{ y_{i,t} < x_{i,t}^{{\prime }} \beta_{i,q} \} }}$ is the usual indicator function. The solution to the quantile regression model is obtained using the programming algorithm suggested by Koenker and D’Orey (1987). The standard errors for the estimated coefficients and the confidence intervals for each parameter can be obtained using the bootstrap method (Hao and Naiman 2007).^5^

There are two major advantages of using quantile regression in this study. First, quantile regression allows inferences regarding the impact of oil market structural shocks conditional on the distribution of the real exchange rate returns. The quantile regression parameters estimate the change in a specified quantile of real exchange rate as a response to a 1-unit structural shock in the oil market. Second, quantile regression has no assumption regarding the distribution of the error terms. This flexibility causes quantile regression estimates to exhibit stronger model robustness compared to those obtained from OLS regression.

## Data and summary statistics

**Fig. 1:**
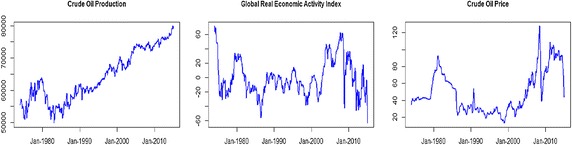
Global oil production, global economic activity and real oil price

In order to identify oil market supply and demand shocks, monthly data are collected on global oil production, global real economic activity, oil price and exchange rates. The global oil production (in thousands of barrels per day) and oil price (in dollars per barrel) are sourced from the US Energy Information Administration. Oil prices are deflated by the US CPI. The global real economic activity index is likely to capture shifts in demand for industrial products in global business markets and is available to download from Lutz Kilian’s homepage. These three data sources cover the sample period from January 1974 to March 2015. Figure 1 illustrates global oil production, the global real economic activity index and the real oil price. Since the 1980s, global oil production has increased, reaching as high as 7927.44 thousand barrels in March 2015. Relative to the dynamics of global oil production, the real oil price does not express the trend of continued increase or decrease.

**Fig. 2:**
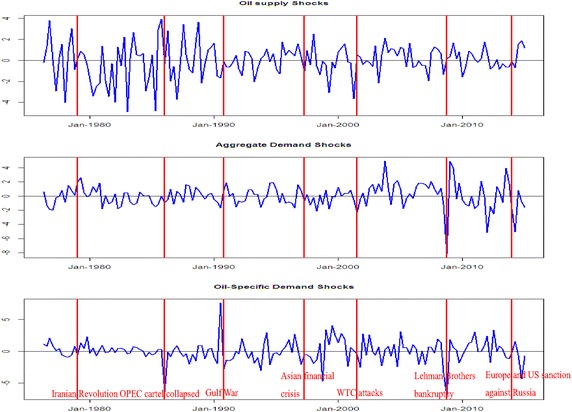
Oil supply shocks, global economic demand shocks and oil demand shocks

Table 1 shows the results of unit root tests and provides ample evidence that each variable in our VAR model is stationary.^6^ Following Kilian (2009), Atems et al. (2015) and Basher et al. (2016), the VAR model Eq. (1) is estimated with 24 lags, i.e. $p = 24$. The evolutions of the identified oil market structural shocks are plotted in Fig. 2. To improve readability, the shocks are aggregated by summing up the shocks in each quarter. The identified oil supply and demand shocks reflect information about the oil market. For example, in the mid to late 1980s, there was a relatively large negative oil-specific demand shock as the OPEC cartel collapsed. The identified aggregated demand shock and oil-specific demand show a decrease in the late 2000s following the global financial crisis.

**Table 1:** ADF unit root tests for the VAR model

	None	With intercept	With trend and intercept
∆*prod*	−24.2046 (0.0000)	−24.2363 (0.0000)	−24.2226 (0.0000)
*rea*	−3.8291 (0.0001)	−3.8450 (0.0027)	−3.8980 (0.0128)
*rpo*	−12.4438 (0.0000)	−12.4312 (0.0000)	−12.4191 (0.0000)

The optimal lag length for the tests was determined using the Schwarz Information Criterion (SIC). The p-values are reported in parentheses

**Fig. 3:**
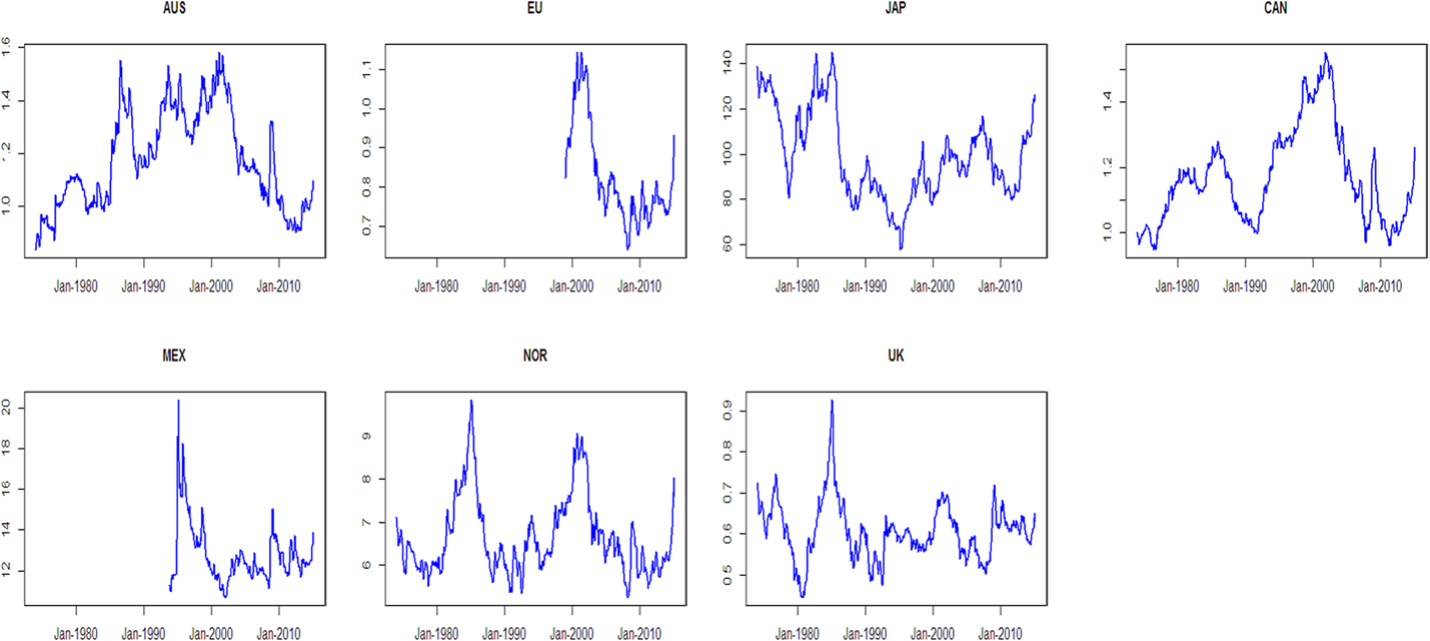
Real exchange rates

The exchange rate data for Australia (AUS), Canada (CAN), the European Union (EU), Japan (JAP), Mexico (MEX), Norway (NOR) and the United Kingdom (UK) are sourced from the St. Louis Federal Reserve’s FRED database. For all countries, the exchange rates are quoted as foreign currency per US dollar. These countries are selected to investigate the impact of oil shocks on real exchange rates because they not only account for the vast majority of market trading in international exchange rates but are also important oil-importing and oil-exporting counties.^7^ Furthermore, the countries represented are both developed and emerging economies, as well as oil-exporting and oil-importing countries, as the effects of oil shocks on exchange rates may vary according to these characteristics. Of these countries, Canada, Mexico, Norway and the United Kingdom are classified as oil exporting countries. Australia, Japan and the European Union are classified as oil-importing countries. The estimation sample period varies by country due to data limitations. For Australia, Canada, Japan, Norway and the United Kingdom, models are estimated over the period from January 1974 to March 2015. For the European Union and Mexico, the sample period is January 2000 to March 2015 and December 1993 to March 2015 respectively. The nominal exchange rates are converted to real exchange rates using the CPI ratio between the two countries. The nominal exchange rates were downloaded from the Federal Reserve Bank of Saint Louis and the CPI data are available from the OECD. Figure 3 shows that each real exchange rate has experienced considerable variability across time. Moreover, these plots of the real exchange rates indicate little evidence of a linear structure. In the quantile regression model, we use monthly real exchange rate returns of each country which are constructed using $r_{t} = 100 \times \ln (p_{t} /p_{t - 1} )$, where $p_{t}$ is the real exchange rate at period $t$.

Table 2 presents descriptive summary statistics for the oil structural shocks and real exchange rate returns. The mean value of each of the three structural shocks is zero, and each structural shock has a standard deviation close to unity. Each of structural shock displays similar variability which measured by difference between the maximum value and minimum value. However, unlike with the oil price shock, the oil supply and demand shocks show negative skewness and excess kurtosis. In real exchange rate returns, the Mexican peso has the greatest amount of variability and the European Union euro has the least. The currencies of the European Union and Japan appear negative values for skewness. The currencies of Australia, Canada and Mexico display excess kurtosis, ranging from 5.7301 to 27.3467. According to the Shapiro–Wilk test, normality of the unconditional distribution was strongly rejected for all structural shock and real exchange rate returns. In order to test for the presence of a unit root against the alternative of a stationary process in the structural shocks and real exchange rate returns, the Augmented Dickey Fuller (ADF) test is conducted. The test result illustrates that in each of the oil structural shocks and real exchange rate returns time is stationary.

**Table 2:** Summary statistics on oil shock and monthly real exchange rate returns

	Supply	Demand	Price	AUS	EU	JAP	CAN	MEX	NOR	UK
Min	−4.4061	−4.7628	−3.5219	−5.9461	−6.7545	−10.9560	−6.4209	−15.7770	−6.0399	−11.1840
Max	3.6987	3.9909	4.8301	15.2569	7.0501	8.2285	11.3194	31.7050	11.7510	7.4383
Median	0.0384	0.0140	0.0299	−0.1368	0.1525	0.1428	0.01095	−0.3407	−0.0560	−0.04538
Mean	0.0000	0.0000	0.0000	0.0545	0.0633	−0.0188	0.0470	0.0803	0.0245	−0.0221
Std. dev	0.9492	0.9474	0.9605	2.4506	2.4723	2.7945	1.4633	3.5369	2.4184	2.4689
Skewness	−0.7651	−0.5716	0.0418	1.3235	−0.1091	−0.4751	0.6452	3.1931	0.3178	0.0256
Kurtosis	4.4061	4.2286	1.9673	5.7301	0.0983	0.9756	7.5366	27.3467	0.7978	1.8912
Norm test. W	0.9311	0.9356	0.9806	0.9303	0.9939	0.9837	0.9445	0.7268	0.9911	0.9833
Norm test. p	0.0000	0.0000	0.0000	0.0000	0.6094	0.0000	0.0000	0.0000	0.0045	0.0000
Unit root test. A	−7.8977	−7.3289	−7.2995	−8.5445	−4.6561	−7.2583	−7.0261	−7.2291	−7.3868	−7.6444
Unit root test. p	0.0000	0.0000	0.0000	0.0000	0.0000	0.0000	0.0000	0.0000	0.0000	0.0000

The ADF unit root tests have been using the model with intercept and linear trend. The optimal lag length for the tests was determined using the Schwarz information criterion (SIC)

## Empirical results

**Fig. 4:**
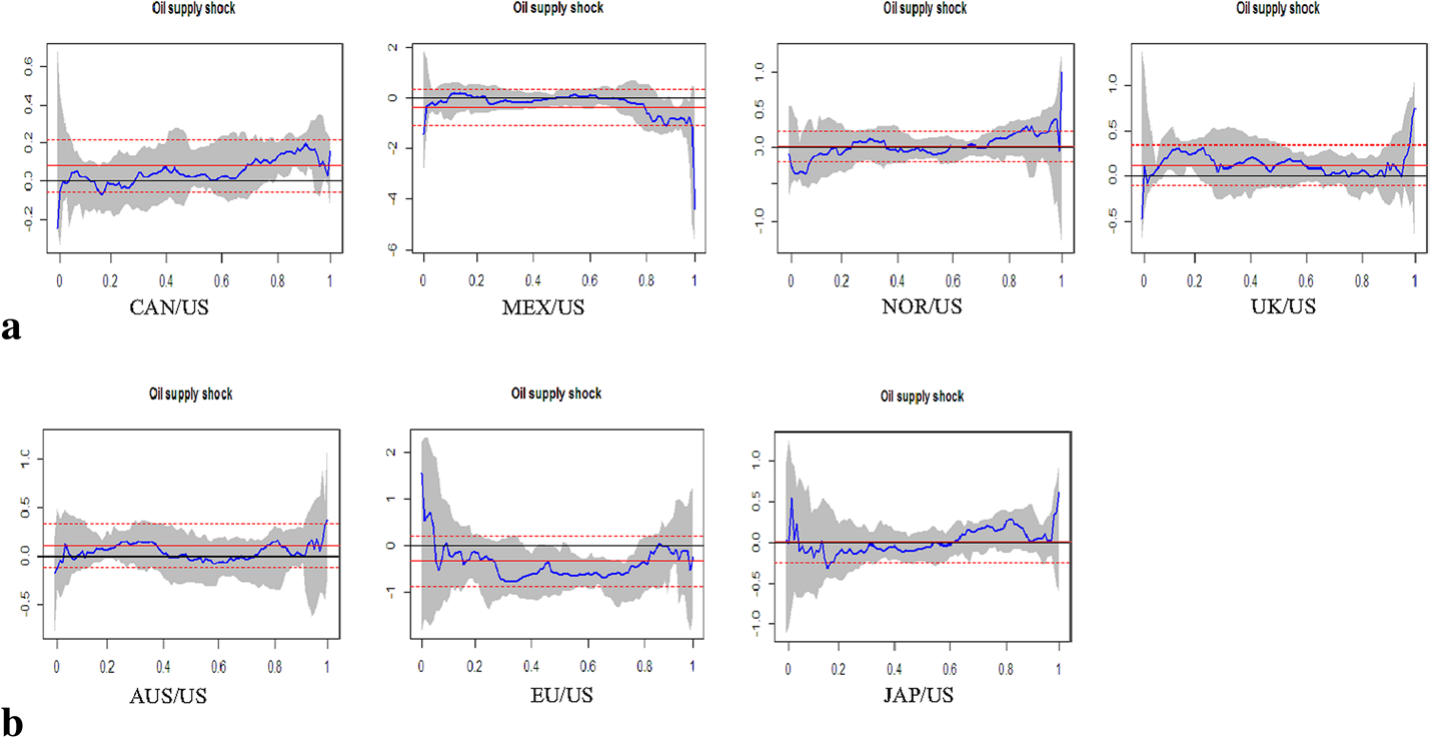
Oil supply shocks on exchange rate returns. **a** Oil-exporting countries. **b** Oil-importing countries

To improve our understanding of heterogeneous impact of oil shocks on real exchange rates, we analyze the determinants of real exchange rate return changes in different quantiles and investigate whether the effects of oil shocks on real exchange rates vary cross different quantiles. We estimate quantile regression for lower quantiles (0.01, 0.05, and 0.1), the central quantile (0.5 quantile), and upper quantiles (0.90, 0.95, and 0.99 quantiles).^8^ These results help us to determine how changes in the oil shocks affect real exchange rates at the tails of the return distribution and indicate what is the difference in the effect of oil shocks on real exchange rates during periods of US dollar appreciation or depreciation. The empirical results of the general impact of crude oil shocks on real exchange rates based on quantile regression (at the 0.5 quantile) and OLS regression estimates are reported in Table 3. The extreme impact based on quantile regression (at the lower and upper quantiles) estimates are shown in Table 4. The empirical results show that parameter estimates vary across the OLS and quantiles. Moreover, the magnitude and direction of coefficients may vary across the quantiles. The estimation outputs for the quantile regressive model are depicted graphically in Fig. 4. We contrast the sequences of the estimated coefficients over all quantiles, together with the corresponding OLS regressive coefficients and their 95 % confidence bands. In this study, following Baur (2013), the sequence of the estimated coefficients is used to capture the structure of oil shocks on exchange rates.

**Table 3:** The general impact of crude oil shocks on real exchange rates

	AUS	EU	JAP	CAN	MEX	NOR	UK
Panel A: OLS regression							
Intercept	0.0292 (0.1088)	0.0728 (0.1697)	−0.0027 (0.1243)	0.0463 (0.0654)	0.1063 (0.2197)	0.0362 (0.1033)	0.0061 (0.1089)
Oil supply shock	0.1037 (0.1155)	−0.3329 (0.2772)	0.0135 (0.1318)	0.0775 (0.0693)	−0.3595 (0.3646)	0.0127 (0.1096)	0.1120 (0.1155)
Aggregate demand shock	−0.3465*** (0.1154)	−0.1703 (0.1429)	0.1886 (0.1315)	−0.2430*** (0.0703)	−0.0114 (0.1959)	−0.1238 (0.1103)	−0.1933* (0.1152)
Oil-specific demand shock	−0.3228*** (0.1150)	−0.2976* (0.1512)	0.0477 (0.1313)	−0.2764*** (0.0690)	−0.2959 (0.2035)	−0.4343*** (0.1091)	−0.2187* (0.1154)
Panel B: Qunatile regression (q = 0.5)							
Intercept	−0.1296 (0.1429)	0.2697 (0.2292)	0.1194 (0.1733)	0.0645 (0.0894)	−0.3210* (0.1885)	0.0172 (0.1471)	0.0266 (0.1524)
Oil supply shock	−0.0467 (0.1540)	−0.5870 (0.3745)	−0.0708 (0.1636)	0.0218 (0.0963)	0.0204 (0.3124)	−0.0541 (0.1355)	0.1323 (0.1704)
Aggregate demand shock	−0.1448 (0.1710)	−0.2315 (0.1930)	0.2911 (0.1790)	−0.0058 (0.1250)	0.1004 (0.2001)	−0.1137 (0.1578)	−0.0056 (0.1893)
Oil-specific demand shock	−0.1190 (0.1567)	−0.4246** (0.2041)	−0.0891 (0.1930)	−0.2394* (0.1143)	−0.0451 (0.1745)	−0.4107** (0.1789)	−0.2696 (0.1783)

The standard errors are reported in parentheses

*^,^ ** and *** denotes coefficients significant at 10, 5 and 1 % level respectively

**Table 4:** The extreme impact of crude oil shocks on real exchange rates (lower quantiles)

	AUS	EU	JAP	CAN	MEX	NOR	UK
Panel A: Qunatile regression (q = 0.01)							
Intercept	−4.8641*** (0.1392)	−5.5807*** (0.2085)	−6.8229*** (0.2293)	−3.6080*** (0.1422)	−5.1912*** (0.3373)	−4.9979*** (0.1361)	−66.1070*** (0.2636)
Oil supply shock	−0.1789 (0.1091)	1.5651*** (0.3406)	0.0354 (0.2322)	−0.2534** (0.1135)	−1.4286*** (0.5286)	−0.0823 (0.1395)	−0.4661*** (0.1477)
Aggregate demand shock	0.4632*** (0.1413)	−0.9422*** (0.1756)	−0.3451* (0.1867)	−0.6068*** (0.0915)	0.1269 (0.2911)	−0.1719 (0.1307)	−1.3451*** (0.1424)
Oil-specific demand shock	−0.3124** (0.1286)	−0.4980*** (0.1857)	0.1802 (0.1788)	−0.1766* (0.1006)	0.0411 (0.2365)	−0.2209** (0.1123)	−0.6308*** (0.1398)
Panel A: Qunatile regression (q = 0.05)							
Intercept	−3.2435*** (0.1447)	−4.5158*** (0.4670)	−4.5083*** (0.2492)	−2.1805*** (0.1186)	−3.4768*** (0.2349)	−3.7680*** (0.2014)	−3.5623*** (0.1741)
Oil supply shock	0.1243 (0.1680)	0.7149 (0.7255)	0.2278 (0.3263)	−0.0010 (0.1106)	−0.1903 (0.3818)	−0.3363* (0.1802)	0.0208 (0.1494)
Aggregate demand shock	−0.1607 (0.1315)	−0.7223** (0.3414)	0.0529 (0.2280)	−0.3136*** (0.0854)	−0.0202 (0.2235)	−0.1524 (0.1947)	−0.2689* (0.1450)
Oil-specific demand shock	−0.3320** (0.1609)	0.0899 (0.3063)	0.7625*** (0.2192)	−0.1149 (0.0995)	−0.0611 (0.2142)	−0.2364 (0.1465)	−0.0066 (0.1436)
Panel A: Qunatile regression (q = 0.10)							
Intercept	−2.6168*** (0.1402)	−2.6674*** (0.2775)	−3.6585*** (0.2409)	−1.6529*** (0.1015)	−2.7916*** (0.2176)	−2.8608*** (0.1833)	−2.9058*** (0.1685)
Oil supply shock	0.0198 (0.1467)	−0.0240 (0.5136)	−0.0331 (0.3030)	0.0192 (0.1006)	−0.1919 (0.3647)	−0.1228 (0.1923)	0.2035 (0.1682)
Aggregate demand shock	−0.1541 (0.1197)	0.1582 (0.2316)	0.2763 (0.2495)	−0.1724** (0.0846)	0.0485 (0.2198)	−0.1176 (0.1777)	−0.2973** (0.1467)
Oil-specific demand shock	−0.1753 (0.1566)	−0.4169 (0.2601)	0.4293** (0.2107)	−0.1170 (0.0945)	−0.3399* (0.1889)	−0.2351 (0.1556)	−0.0864 (0.1448)

The standard errors are reported in parentheses

*^,^ ** and *** denotes coefficients significant at 10, 5 and 1 % level respectively

### The general impact of oil shocks on exchange rates

The OLS regression can capture the average impact of oil shocks on exchange rates and the quantile regression (at the 0.5 quantile) can represent the central impact of oil shocks on exchange rates. Although the experiment results derived from OLS and quantile regression (at the 0.5 quantile) scarcely differ in many cases we still consider that both the experiment results can present the general impacts of oil shocks on exchange rates. As shown in Table 2, all of the time series are skewed and have a non-normal distribution, which implies that the quantile regressive results have stronger robustness compared with the OLS estimation.

Table 3 describes the general impacts of oil structural shock on exchange rates. For oil supply shocks, the empirical results derived from both OLS regressive and quantile regressive (at the 0.5 quantile) estimations indicate that oil supply shocks have no statistically significant general impacts on real exchange rates in both oil-importing and oil-exporting countries. The coefficients estimated by quantile regression (at the 0.5 quantile) on global aggregate demand shocks variable have no statistically significant central effects on real exchange rates in any country, which is different from the OLS regressive results.^9^ Oil-specific demand shocks have negative and statistically significant general impacts on real exchange rates for oil-exporting countries (Canada, Norway and United Kingdom) and oil-importing countries (Australia and the European Union). Only for oil-importing Japan, the oil-specific demand shock has a positive impact, but it has no statistical significance.

We find that oil supply shocks have no significant effects on exchange rates, while the oil demand shocks show significant effects on exchange rates although there is no systematic pattern. For oil-exporting countries (Canada, Norway, and the UK), an oil demand shock leads to significant appreciation pressures. This finding is in line with the empirical results of Buetzer et al. (2012), who find that oil exporters experience significant appreciation pressures following an oil demand shock. For oil-exporting countries, a positive oil-specific demand shock generally leads to an improvement of the trade balance and subsequently to an appreciation of the local currency. However, as an oil-exporting country, Mexico does not show significant appreciation pressure. A plausible explanation is that depending on the share of commodity exports in a country’s total exports, central banks have incentives to actively counter appreciation pressure by accumulating foreign exchange reserves. This effect may lessen a systematic appreciation of exchange rates in oil-exporting countries in response to a positive oil demand shock. For oil-importing economies (Australia and the EU), we find the counterintuitive empirical result that a positive oil demand shock causes the oil importer’s real exchange rate to appreciate, which implies that a deterioration in the oil components of their trade balance is not offset by an improvement in the non-oil trade balance (Basher et al. 2016). Furthermore, Cashin et al. (2014) indicate that various country-specific differences in monetary and fiscal policies, exchange rate regimes, and product and labor market rigidities will affect the responses of exchange rates to various oil shocks.

### The extreme impact of oil shocks on exchange rates

For a significant US dollar depreciation (at lower quantiles), the extreme impacts of oil supply and demand shocks on exchange rates are shown in Table 4. For oil-exporting countries, the estimated coefficients on oil supply shock variables are negative and statistically significant for Canada, Mexico and the UK at the 0.01 quantile, and for Norway at the 0.05 quantile. While for oil-importing countries, oil supply shocks only have positive and significant impacts on exchange rate returns in the EU. These results can be easily explained. The oil supply shocks possibly reflect the discovery of new oil fields, better extraction technologies or a decline in OPEC’s control over the oil supply. Following an oil supply shock that alters the oil balance, the real exchange rate moves to ensure an offsetting adjustment in the non-oil trade balance in order to stabilize net foreign assets. Therefore, a positive oil supply shock has negative impacts (US dollar depreciation) on exchange rate returns in oil-exporting countries while having positive impacts (US dollar appreciation) in oil-importing countries.

Aggregate demand shocks have negative and statistically significant impacts on exchange rate returns in oil-exporting countries (Canada and the UK) at three lower quantiles, which implies that positive aggregate demand shocks bring about an appreciation of the local currency relative to the US dollar. The aggregate demand shocks have statistically significant effects on exchange rate returns in oil-importing countries at the 0.01 quantile. For Japan and the European Union, the shock effects are negative, whereas for Australia they are positive. Aggregate demand shocks reflect typical short-run macroeconomic multiplier effects over the business cycle from aggregate demand stimulation. A global aggregate demand-driven shock would affect oil-exporting countries’ currencies both through a change in the oil price and through a change in demand for other goods they export. Therefore, the aggregate demand shocks lead to a negative impact (US dollar depreciation) on exchange rate returns in oil-exporting countries. However, for oil-importing countries, the impact on the effective exchange rate may differ due to compensatory effects through trade and asset channels vis-à-vis other main trading partners.

For oil-exporting countries, the oil-specific demand shocks have a negative and statistically significant impact on exchange rates in Canada, Norway and the United Kingdom at the 0.01 quantile, and in Mexico at the 0.1 quantile. This is consistent with prior expectations and empirical evidence that rising oil prices cause an appreciation of an oil-exporting country’s currency (US dollar depreciation). For oil-importing countries, oil-specific demand shocks have negative and significant impact on exchange rate returns in Australia and the EU while they have a positive and significant impact in Japan. Depending on the share of commodity imports in a country’s total imports, central banks have incentives to actively ensure the external sustainability of oil-importing countries by reducing foreign exchange reserves. This effect may mitigate the systematic effect of exchange rates in oil-importing countries in response to oil-specific demand shocks.

For a significant US dollar appreciation, Table 5 presents the extreme impact of oil structural shocks on exchange rates at the 0.90, 0.95 and 0.99 quantiles. Oil supply shocks have a significant impact on real exchange rates in oil-exporting countries. For Canada, Norway and the United Kingdom, the shock effects are positive, but they are negative for Mexico. These results may seem surprising because oil supply shocks have positive shock effects on exchange rate returns (US dollar appreciation) in oil-exporting countries, i.e., depreciate oil-exporters’ currency, which is inconsistent with the previous theory. A plausible explanation is that in the upper quantiles of return distributions, US dollar appreciation, market participants’ risk aversion and the US dollar’s dominance as a safe-haven currency will lead to self-reinforcing effects of the US dollar and result in US dollar appreciation, which may offset the negative effects of oil shocks. In oil-importing countries-Australia and Japan-the estimated coefficient on the oil supply shock is positive and statistically significant. The aggregate demand shocks have a negative and significant impact on real exchange rates in Canada and Norway. In the oil importing countries (Australia and Japan), aggregate demand shocks have a significant impact on real exchange rates. For Australian a positive aggregate demand shock tends to dampen US dollar appreciation (negative shock effects), implying an appreciation of Australia currency. However, for Japan, the shock effect is positive, thus indicating US dollar appreciation; hence, there it has a tendency to be amplified by a positive aggregate demand shock. The estimated coefficients on the oil demand shock variables are negative and statistically significant for the oil-exporting countries of Canada, Mexico and Norway at the three upper quantiles. For oil-importing countries (Australia and the EU), oil-specific shocks have negative and significant impacts on exchange rate returns, while they have positive and significant impacts in Japan. Although the result for Japan is unexpected, Japan’s current account surplus and reliance on nuclear energy could explain this phenomenon.

**Table 5:** The extreme impact of crude oil shocks on real exchange rates (upper quantiles)

	AUS	EU	JAP	CAN	MEX	NOR	UK
Panel A: Qunatile regression (q = 0.90)							
Intercept	2.7003*** (0.1773)	2.9698*** (0.2586)	3.2154*** (0.1766)	1.6453*** (0.0934)	3.3257*** (0.3981)	2.7998*** (0.1636)	2.8933*** (0.1769)
Oil supply shock	−0.0034 (0.1719)	−0.1478 (0.3685)	0.0325 (0.1807)	0.1926*** (0.0732)	−0.5486 (0.5324)	0.1291 (0.1659)	0.0686 (0.1766)
Aggregate demand shock	−0.4645** (0.2132)	0.0717 (0.1959)	0.2370 (0.1968)	−0.1380 (0.0964)	0.1376 (0.2774)	−0.2524 (0.2286)	−0.1448 (0.1829)
Oil-specific demand shock	−0.4333*** (0.1514)	0.0693 (0.2375)	−0.0865 (0.1981)	−0.1607* (0.0915)	−0.6608** (0.3235)	−0.6249*** (0.1733)	−0.2644 (0.2008)
Panel A: Qunatile regression (q = 0.95)							
Intercept	4.2069*** (0.3005)	4.1133*** (0.3106)	4.1003*** (0.1967)	2.2198*** (0.1176)	4.5984*** (0.4934)	3.5201*** (0.1712)	4.0087*** (0.2287)
Oil supply shock	−0.2627 (0.2679)	−0.1195 (0.4031)	−0.0086 (0.2115)	0.0745 (0.0984)	−0.5407 (0.5800)	0.3247** (0.1574)	0.1688 (0.2376)
Aggregate demand shock	−0.8399*** (0.2909)	−0.1878 (0.1883)	0.2838 (0.2335)	−0.1941* (0.1051)	0.0829 (0.2491)	−0.1299 (0.2332)	−0.2364 (0.1888)
Oil-specific demand shock	−0.7173*** (0.2208)	−0.3921* (0.2174)	0.0970 (0.2433)	−0.3771*** (0.1018)	−0.7807** (0.3497)	−0.6269*** (0.1628)	−0.2669 (0.2304)
Panel A: Qunatile regression (q = 0.99)							
Intercept	6.5155*** (0.2983)	5.3645*** (0.3142)	5.5931*** (0.1423)	3.2089*** (0.0961)	10.6930*** (0.8447)	5.9670*** (0.2202)	5.1933*** (0.1544)
Oil supply shock	0.3677* (0.1905)	−0.4861 (0.5133)	0.6137*** (0.1109)	0.1589** (0.0717)	−4.4077*** (0.9849)	1.0051*** (0.1430)	0.7489*** (0.1345)
Aggregate demand shock	−1.2592*** (0.1976)	0.0072 (0.2646)	0.9376*** (0.1229)	−0.4897*** (0.0896)	0.4345 (0.2917)	−0.6062*** (0.1652)	−0.1874 (0.1252)
Oil-specific demand shock	−0.5733*** (0.1706)	−0.5541** (0.2799)	0.4135*** (0.1406)	−0.4697*** (0.0722)	−1.9372*** (0.4870)	−0.4643*** (0.1408)	−0.0042 (0.1363)

The standard errors are reported in parentheses

*^,^ ** and *** denotes coefficients significant at 10, 5 and 1 % level respectively

Compared with the general impacts of oil structural shocks on exchange rates, the extreme impacts at the lower and upper quantiles express more significance statistically. For oil supply shocks, the general impacts tend to not be statistically significantly distinguishable from zero. However, the estimated coefficients on the oil supply variable in quantile regression at extreme quantiles are significant negative for oil-exporting countries. Moreover, the oil supply shocks have positive and significant impacts on exchange rate returns in Australia and Japan at the upper quantiles and negative and significant impacts in the EU at the lower quantile. Not surprisingly, the lower quantile levels represent significant US dollar depreciation. A significant US dollar depreciation should lead to a situation in which consumers with appreciated currencies find oil less expensive, thus increasing their demand for oil, which would induce an improvement in the real trade balance and current account surplus for oil-exporting economies; it would also induce a deterioration of the terms of trade and a current account deficit for oil-importing economies. This may amplify the impact of the oil supply on exchange rates and make the impact of shocks more significant statistically. Taken together, there is strong evidence that important information is essentially hidden behind the simple OLS coefficients.

### The structure of oil shocks on exchange rates

Figure 4 reveals a noticeable difference in the effect of oil supply shocks on real exchange rates across various quantiles. The sequence of the estimated coefficients can express the structure of oil supply shocks on real exchange rates. Panel A reveals the shock structure of oil supply on oil exporting countries. The structure of shock tends to be inverse S-shaped. Unlike the median quantile, in the lower and upper quantiles, the estimated coefficient on the oil supply shock variables tends to be statistically distinguishable from zero. This implies that for significant US dollar depreciations, the shock is mostly negative, whereas for significant US dollar appreciations it is mostly positive. This pattern is clearly apparent for Canada, Norway and the United Kingdom. However, for Mexico, the shock structure of oil supply tends to be inverse U-shaped. In both the lower and upper quantiles, the oil supply shocks have negative and statistically significant impacts on its real exchange rate. The structure of oil supply shocks on real exchange rates in oil-importing countries is shown in the Panel B. These shock structures tend to be U-shaped in most cases. At the lower quantiles, oil supply shocks have positive and statistically significant effects on both Japan and the European Union. However, at the upper quantiles, only for Japan are the oil supply shocks positive and statistically significant. Overall, the effects of oil supply shocks on real exchange rates have heterogeneous impacts on different countries and across various quantiles. In particular, for significant US dollar depreciation and appreciation, the impact of oil supply shocks on exchange rates is significantly distinguishable from zero.

**Fig. 5:**
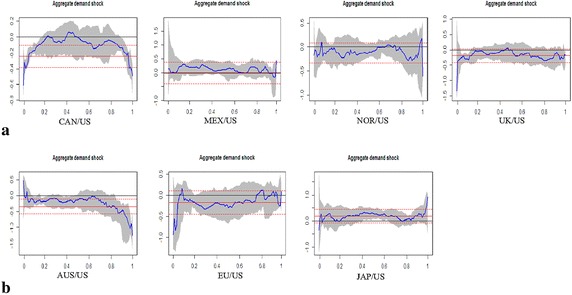
Aggregate demand shocks on exchange rate returns. **a** Oil-exporting countries. **b** Oil-importing countries

Figure 5 provides complete structures of aggregate demand shocks on real exchange rates. For Canada, the structures of aggregate demand shocks tend to be inverse U-shaped, indicating that significant negative shock effects tend to prevail in both the upper and lower quantiles. For the other oil-exporting countries, on the other hand, the structure of aggregate demand shock shows different patterns. For Mexico and Norway, there is virtually no evidence for statistically significant shock effects, and for the United Kingdom, the negative shock effect tends to prevail in both the upper and lower quantiles. However, the statistical significance is only apparent for the lower quantiles. Now we consider the structures of aggregate demand shocks on importing countries. For Australia, the significant positive shock effect tends to prevail in lower quantiles and the significant negative shock effect tends to prevail in upper quantiles. The particular structure of the shock tends be S-shaped. The pattern of oil demand shocks on Japan’s real exchange rate is simply the reverse of the S-shape. This implies that the significant negative shock effect tends to be present in lower quantiles and the significant positive shock effect tends to present in the upper quantiles. For the United Kingdom, the estimated coefficient on the aggregate demand shock variable is negative and statistically significant in the lower quantiles of the exchange rate return distribution (US dollar depreciations), which indicates that a positive oil demand shock tends to dampen US dollar depreciations.

**Fig. 6:**
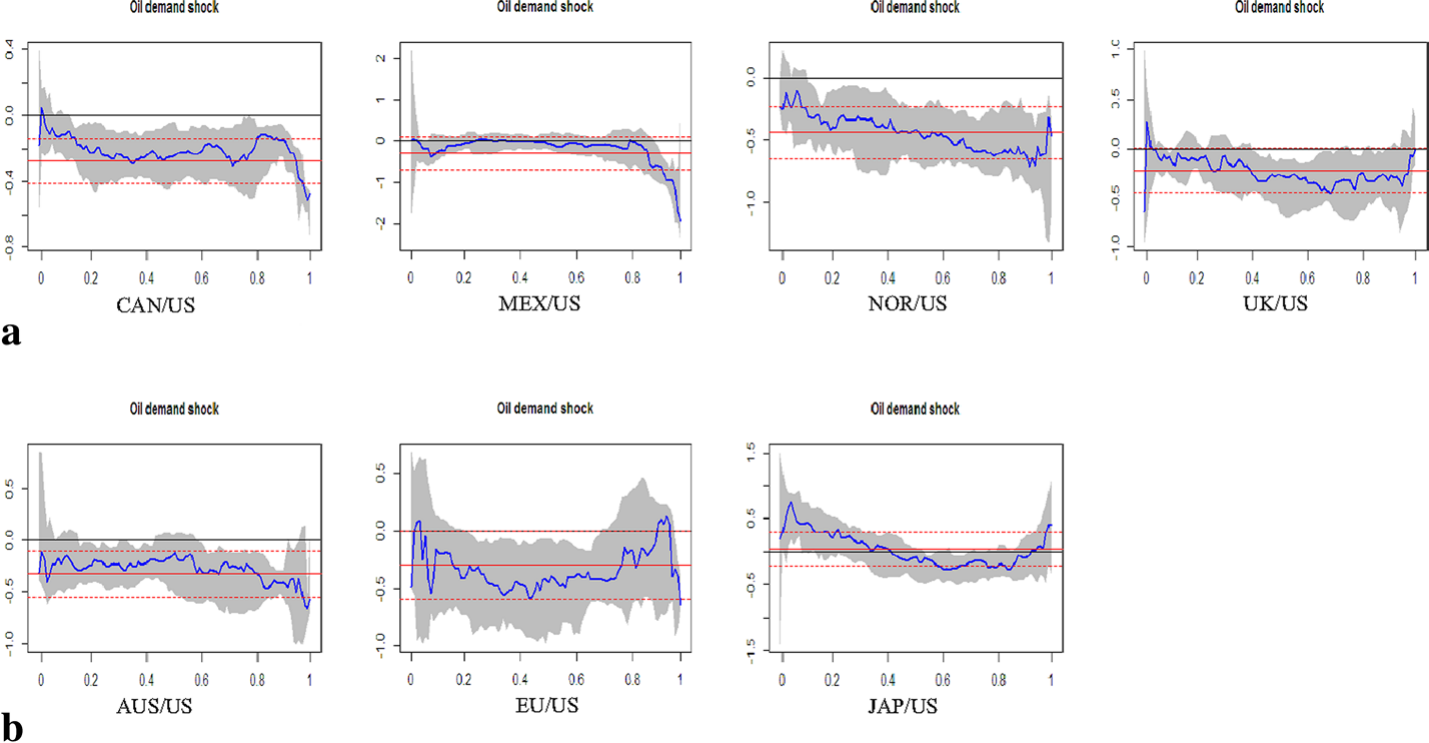
Oil-specific demand shocks on exchange rate returns. **a** Oil-exporting countries. **b** Oil-importing countries

Figure 6 also illustrates the structures of shock of oil-specific demand on real exchange rates. For oil-exporting countries, the oil-specific demand shocks have negative and statistically significant impacts across various quantiles with the exception of Mexico, where the statistical significance is only apparent for the upper quantiles. It is obvious that the oil-specific shock effects at the upper quantiles are larger than at the lower quantiles. This implies that although a positive oil price shock tends to amplify US dollar depreciations and dampen US dollar appreciations, but the extent of dampening is more than the extent of amplification. In oil-importing countries (Australia and the EU), oil-specific demand shocks also have significant negative impact across almost all quantiles. For Japan, however, the particular structure of the oil price shock tends be U-shaped. The significant positive shocks tend to prevail in both the lower and upper quantiles. This indicates that a positive oil price shock tends to amplify US dollar appreciations and dampen US dollar depreciations.

In addition, the parameter heterogeneity test can be examined using inter-quantile tests that are developed to examine whether the differences along the estimated coefficients are statistically significant across quantiles. More specifically, following Koenker and Bassett (1982), Wald tests are performed to check for slope equality across quantiles.^10^ To save space, we only present the results of whether the model in the lower quantile (at the 0.01 quantile) is the same as the median quantile (at the 0.5 quantile) and in the upper quantile (at the 0.99 quantile).^11^ The results are reported in Table 6. The test of equality of the coefficients between the lower quantiles and the upper quantile reject the hypothesis of parameter homogeneity with the exception of a few cases. In summary, we can conclude that it is important to take into account the return distribution heterogeneity to investigate the impacts of oil structural shocks on exchange rate returns.

**Table 6:** Wald tests for equality of slopes (0.01 against 0.5 and 0.99 quantiles)

Country	Structural oil price	Against the 0.5 quantile		Against the 0.99 quantile	
		Test statistic	p value	Test statistic	p value
AUS	Oil supply shock	0.6217	0.4306	6.2482	0.0126**
	Aggregate demand shock	8.4129	0.0038***	50.6984	0.0000***
	Oil-specific demand shock	0.9698	0.3249	1.5048	0.2202
EU	Oil supply shock	14.1023	0.0001***	9.9400	0.0017***
	Aggregate demand shock	5.2877	0.0220**	14.4830	0.0001***
	Oil-specific demand shock	0.0315	0.8592	0.3250	0.5689
JAP	Oil supply shock	0.2050	0.6507	5.0676	0.0246**
	Aggregate demand shock	6.4541	0.0112**	33.0522	0.0000***
	Oil-specific demand shock	1.1595	0.2818	1.0574	0.3040
CAN	Oil supply shock	3.5670	0.0592*	9.8332	0.0017***
	Aggregate demand shock	16.5285	0.0000***	0.8439	0.3585
	Oil-specific demand shock	0.1873	0.6652	5.7140	0.0170**
MEX	Oil supply shock	6.2761	0.0125**	7.1768	0.0076***
	Aggregate demand shock	0.0064	0.9361	0.5681	0.4513
	Oil-specific demand shock	0.0991	0.7530	13.5531	0.0002***
NOR	Oil supply shock	0.0237	0.8778	29.5530	0.0000***
	Aggregate demand shock	0.0895	0.7648	4.2635	0.0392**
	Oil-specific demand shock	0.8863	0.3467	1.8353	0.1758
UK	Oil supply shock	7.0492	0.0080***	37.3880	0.0000***
	Aggregate demand shock	35.3164	0.0000***	37.6230	0.0000***
	Oil-specific demand shock	2.9442	0.0865*	10.4730	0.0012***

The bootstrap method is employed to obtain the variance-covariance matrix for this estimator used in Wald test

*, ** and *** denotes coefficients significant at 10, 5 and 1 % level respectively

## Conclusions

The objective of this study is to examine the sensitivity of exchange rates to oil shocks and the conditional distribution of exchange rate return. This is an important topic because a large exchange depreciation or appreciation can alter a country’s terms of trade and current account balances, which are the main transmission channels for oil shocks. Concerning the effects of oil shock variables on different parts of the exchange rate distribution can help us obtain a more complete picture of the factors affecting exchange rates.

Our approach is to estimate the heterogeneous effects of oil shocks on exchange rates using a quantile regression model. This approach has the advantage of capturing the distributional heterogeneity of exchange rate responses that linear models would be unable to detect. Using this methodology, we are able to assess the determinants of exchange rate change across the conditional distribution, with a particular focus on significant US dollar depreciation and appreciation. Moreover, the oil supply shocks, aggregate demand shocks, and oil-specific demand shocks are included in our quantile regression models. This provides a more complete understanding of how the oil market affects real exchange rates.

There are several interesting findings that stem from the present analysis. The impact of oil shocks on exchange rates presents distributional heterogeneity across various quantiles. The estimated coefficients on the oil shock variables at the lower and upper quantiles are significantly distinguishable from zero, which indicates that a significant US dollar depreciation and appreciation tends to heighten the responses of exchange rates to oil shocks. Although the impacts of oil price shocks on exchange rates have no systematic pattern across oil-importing and oil-exporting countries, we detect significant negative effects on exchange rate responses to oil demand shocks in oil-exporting countries. The result is robust for general and extreme shock effects, and it demonstrates that the oil demand shocks will lead to significant appreciation pressures in oil-exporting countries.

Our results offer some rich and useful information about the relationship between oil price shocks and exchange rate returns. First our results show that the impact of oil price shocks on exchange rate returns is heterogeneous across the return distribution, which helps explain the dynamics of exchange rates and provides an understanding of a mixed oil prices-exchange rates relationship. Second, our results reveal that a significant US depreciation and appreciation will heighten the effects of oil shocks on exchange rate returns, which can offer valuable advices to investors and decision-makers who are interested in the oil price-exchange rate relationship. Third, our results indicate that oil demand shocks are an important factor in exchange rate configurations in oil-exporting countries.

## Footnotes

1 First, the impact of oil price shocks on exchange rates is mainly transmitted by changes in the terms of trade, albeit in different ways to impact oil-exporting and oil-importing countries (e.g., Backus and Crucini 2000; Chen and Rogoff 2003; Cashin et al. 2004; Buetzer et al. 2012; Fratzscher et al. 2014). For oil-importing countries, an increase in oil prices generally leads to deterioration of the trade balance and subsequently to a depreciation of the local currency. On the other hand, for oil-exporting countries, an increase in oil prices often causes a positive terms of trade shock, and a positive terms of trade shock may eventually lead to a Dutch Disease phenomenon by driving up the price of the non-tradable goods and an appreciation of the local currency. Another important channel of transmission of oil price shocks to exchange rates is the wealth effects channel. According to this view, a rise in oil prices is associated with wealth transfers from oil-importers to the oil exporting economy, which leads to a real depreciation (appreciation) of the exchange rates of oil importers (oil exporters) through current account imbalances and portfolio reallocation (e.g., Bodenstein et al. 2011; Rasmussen and Roitman 2011; Buetzer et al. 2012; Fratzscher et al. 2014).

2 The “export selection effect” indicates that the presence of fixed costs to export generates a selection mechanism through which only the best performers are able to export. This implies that a very large share of aggregate exports is made by a small portion of high-performance and large firms. Hence, exporters are firms that optimally choose to partially absorb exchange rate movements in their markups.

3 Kuck et al. (2015) define the exchange rate as US dollar per foreign currency. Therefore, a negative exchange rate return implies an appreciation of the US dollar (left tail of the return distribution) and a positive exchange rate return implies a depreciation of the US dollar (right tail of the return distribution).

4 Note that we make the assumption that the oil structural shock variables arising from Eq. (3) are pre-determined. This is consistent with Kilian (2009). In addition, Atems et al. (2015) and Basher et al. (2016) used the two-stage method to investigate the reaction of exchange rates to oil price shocks.

5 The bootstrap method is a Monte-Carlo method. If we wish to estimate standard errors of quantile regression parameter estimates $\beta_{i,q} = (\beta_{i,0,q} ,\beta_{i,1,q} ,\beta_{i,2,q} ,\beta_{i,3,q} )^{{\prime }}$, which are estimated based on data consisting of sample covariate-response pairs $(x_{i,t} ,R_{i,t} ),t = 1, \ldots ,T$, where $x_{i,t} = (1,\,{\kern 1pt} \varepsilon_{t}^{s} ,\,\varepsilon_{t}^{d} ,\,\varepsilon_{t}^{p} )^{{\prime }}$. We first draw samples of size $T$ with replacement from these pairs. If the number of resample denoted by $M$, we can obtain $M$ bootstrap samples $(x_{i,t} ,R_{i,t} )_{1} , \ldots ,(x_{i,t} ,R_{i,t} )_{M} ,t = 1, \ldots ,T$. Second, for each bootstrap sample, we estimate the parameters $\hat{\beta }_{i,q}^{1} , \ldots ,\hat{\beta }_{i,q}^{M}$. Third, we can estimate the standard error of coefficient estimate $\hat{\beta }_{i,q}$ by taking the standard deviation of the $M$ bootstrap estimates $\hat{\beta }_{i,q}^{1} , \ldots ,\hat{\beta }_{i,q}^{M}$.

6 The three variables in our VAR model follow stationary processes at the 1 % significance level for all cases, whether an intercept and linear trend are included or not, except for global real economic activity where the “with trend and intercept only” case is supported at the 5 % level.

7 These countries have already been empirically investigated regarding the dependence structure between oil and exchange rates (see, e.g., Reboredo 2012; Reboredo et al. 2014). In addition, some large oil exporters (e.g., countries in the Middle East) and a large oil importer (China) are excluded from our analysis because they have no flexible exchange rates (their exchange rates fixed to the US dollar).

8 In principle, the estimation can be performed at any quantile of output for conciseness we present the baseline results for just seven quantiles of output. This choice of quantiles is made to ensure the readability of the presentations and to focus the discussion on the most interesting aspect. More complete results are shown in Fig. 4.

9 The results derived from OLS regression show that the global aggregate demand shocks have negative and statistically significant average effects on real exchange rates for oil-exporting economies (Canada and the United Kingdom) and an oil-importing country (Australia). Overall, for four of the seven countries studied, aggregate demand shocks have no statistically significant impact on real exchange rates.

10 For example, the Wald test is also used by Lamarche (2011) and Flores et al. (2014).

11 Full results of the Wald tests can be obtained from the authors upon request.
